# Access to Mental Health Care during the First Wave of the COVID-19 Pandemic in Italy: Results from the COMET Multicentric Study

**DOI:** 10.3390/brainsci11111413

**Published:** 2021-10-26

**Authors:** Giulia Menculini, Alfonso Tortorella, Umberto Albert, Claudia Carmassi, Giuseppe Carrà, Francesca Cirulli, Bernardo Dell’Osso, Mario Luciano, Maria Giulia Nanni, Maurizio Pompili, Gabriele Sani, Umberto Volpe, Andrea Fiorillo, Gaia Sampogna

**Affiliations:** 1 Department of Psychiatry, University of Perugia, Piazza Lucio Severi 1, 06132 Perugia, Italy; alfonso.tortorella@unipg.it; 2 Department of Medicine, Surgery and Health Sciences, University of Trieste, 34128 Trieste, Italy; ualbert@units.it; 3 Department of Mental Health, Psychiatric Clinic, Azienda Sanitaria Universitaria Giuliano-Isontina—ASUGI, 34128 Trieste, Italy; 4 Department of Clinical and Experimental Medicine, University of Pisa, 56126 Pisa, Italy; ccarmassi@gmail.com; 5 Department of Medicine and Surgery, University of Milan Bicocca, 20126 Milano, Italy; giuseppe.carra@unimib.it; 6 Center for Behavioral Sciences and Mental Health, National Institute of Health, 00161 Rome, Italy; francesca.cirulli@iss.it; 7 Department of Biomedical and Clinical Sciences “Luigi Sacco”, University of Milan, 20157 Milan, Italy; bernardo.dellosso@unimi.it; 8 Department of Health Sciences, Aldo Ravelli Center for Neurotechnology and Brain Therapeutic, University of Milan, 20142 Milan, Italy; 9 Department of Psychiatry, University of Campania Luigi Vanvitelli, Largo Madonna delle Grazie, 80138 Naples, Italy; mario.luciano@unicampania.it (M.L.); andrea.fiorillo@unicampania.it (A.F.); gaia.sampogna@unicampania.it (G.S.); 10 Department of Biomedical and Specialty Surgical Sciences, Institute of Psychiatry, University of Ferrara, 44121 Ferrara, Italy; nnnmgl@unife.it; 11 Department of Neurosciences, Mental Health and Sensory Organs, Faculty of Medicine and Psychology, Sapienza University of Rome, 00185 Rome, Italy; maurizio.pompili@uniroma1.it; 12 Department of Neuroscience, Section of Psychiatry, University Cattolica del Sacro Cuore, 00168 Rome, Italy; gabriele.sani@unicatt.it; 13 Department of Psychiatry, Fondazione Policlinico Agostino Gemelli IRCCS, 00168 Rome, Italy; 14 Clinical Psychiatry Unit, Department of Clinical Neurosciences, Università Politecnica delle Marche, 60121 Ancona, Italy; u.volpe@staff.univpm.it

**Keywords:** COVID-19, lockdown, access to care, mental health services, psychiatric care, stress, loneliness

## Abstract

The COVID-19 pandemic represents an unprecedented public health emergency, with consequences at the political, social, and economic levels. Mental health services have been called to play a key role in facing the impact of the pandemic on the mental health of the general population. In the period March–May 2020, an online survey was implemented as part of the Covid Mental Health Trial (COMET), a multicentric collaborative study carried out in Italy, one of the Western countries most severely hit by the pandemic. The present study aims to investigate the use of mental health resources during the first wave of the pandemic. The final sample consisted of 20,712 participants, mainly females (*N* = 14,712, 71%) with a mean age of 40.4 ± 14.3 years. Access to mental health services was reported in 7.7% of cases. Among those referred to mental health services, in 93.9% of cases (*N* = 1503 subjects) a psychological assessment was requested and in 15.7% of cases (*N* = 252) a psychiatric consultation. People reporting higher levels of perceived loneliness (OR 1.079, 95% CI 1.056–1.101, *p* < 0.001), practicing smart-working (OR 1.122, 95% CI 0.980–1.285, *p* = 0.095), using avoidant (OR 1.586, 95% CI 1.458–1.725, *p* < 0.001) and approach (OR 1.215, 95% CI 1.138–1.299, *p* < 0.001) coping strategies more frequently accessed mental health services. On the other hand, having higher levels of perceived social support (OR 0.833, 95% CI 0.795–0.873, *p* < 0.001) was associated with a reduced probability to access mental health services. The COVID-19 pandemic represents a new threat to the mental health and well-being of the general population, therefore specific strategies should be implemented to promote access to mental healthcare during the pandemic and afterwards.

## 1. Introduction

The COVID-19 pandemic represents an unprecedented public health emergency, with consequences at the political, social, and economic levels [1]. Since the pandemic onset, mental health has been seriously affected [2,3] due to the fear of contagion, social isolation, and lockdown measures [4]. In particular, home confinement and quarantine have led to prolonged separation from relatives and loved ones [5,6], contributing to increasing the levels of distress not only for people with pre-existing mental disorders, but also for those affected by COVID-19, healthcare professionals, and the general population as a whole [7,8,9,10,11]. Furthermore, the SARS-CoV-2 also presents neurotropism and may thus directly trigger possible neuropsychiatric manifestations [12,13,14]. The effects of the COVID-19 pandemic on mental health include the rise of anxiety and depressive symptoms, somatic complaints [6,15,16], disruption of circadian rhythms, with frequent sleep disorders [17], phobias, cognitive deficits, and traumatic experiences.

In this scenario, mental health services have been called to play a key role in order to support not only people with mental disorders, but also “new users” from the general population exposed to the impact of the pandemic [4,18,19]. As a direct consequence of the pandemic and its related containment measures, a substantial reorganization of mental health care has been needed, with the implementation of telemedicine and at-distance consultation [20,21,22,23]. Indeed, remote psychiatric consultations and online psychotherapies have been rapidly implemented, which have been found to be effective [24,25,26]; however, the use of web-based interventions poses major challenges due to disparities in internet access, abilities in using technology, and privacy concerns [21,27,28,29].

During the first weeks of the pandemic, a significant reduction in accessing emergency rooms [30,31,32] and in psychiatric hospitalization rates [31,33] was documented in both the United States and European countries, including Italy. Moreover, a mean decrease of 37% in overall access to healthcare has been reported worldwide, both in outpatient visits and inpatient admissions to different healthcare services [34,35]. The reduced access rate to healthcare could be due to the fear of being infected and to the practical difficulties in reaching services (i.e., due to quarantine or home confinement) [19,21]. Furthermore, in general hospitals, health professionals were redistributed due to special needs associated with the creation of COVID-19-dedicated services [36]. In particular, for mental health services, the reduction in access rate has been more pronounced, also due to stigma and discriminating behaviors [37,38], which got even worse during the pandemic [39,40]. Stigma was listed among the main stressors for subjects suffering from psychiatric disorders [41], but it also affected the general population [5]. It is well known that stigma significantly limits access to care, as a consequence of reduced self-esteem, poor sense of empowerment, and low illness acceptance [42,43,44].

A few studies have assessed the use of mental health resources, especially during the first wave of the pandemic. Most evidence relies on qualitative reports from populations of service users or from data coming from limited settings, i.e., emergency departments [45,46,47,48]. An in-depth description of the use of mental health services during the COVID-19 pandemic is necessary in order to delineate new pathways to care and to develop supportive interventions for the general population [11,49].

Based on these premises, the aims of the present study were: (1) to evaluate the use of mental health resources during the first pandemic wave among the Italian general population; (2) to identify factors associated with access to mental health services. We expect to find an association between referrals to mental health resources during the pandemic and both environmental factors (i.e., financial consequences of COVID-19) and psychopathological features (i.e., presence of a previous mental disorders, insomnia, and depressive symptoms).

## 2. Materials and Methods

### 2.1. Study Design and Procedures

The present study is part of the COMET trial, a multicenter project coordinated by the University of Campania “Luigi Vanvitelli” (Naples). Nine university sites participated in the study, namely Università Politecnica delle Marche (Ancona), University of Ferrara, University of Milan Bicocca, University of Milan “Statale”, University of Perugia, University of Pisa, Sapienza University of Rome, “Catholic” University of Rome, University of Trieste. The Center for Behavioral Sciences and Mental Health of the National Institute of Health in Rome also provided support in the dissemination and implementation of the project in accordance with the clinical guidelines of the National Institute of Health for managing the effects of the COVID-19 pandemic. The study has been approved by the Ethical Review Board of the coordinating center (protocol number: 0007593/i).

The COMET trial includes three different phases, which have already been described elsewhere [6,40]. The survey was carried out between March and May 2020, and it was disseminated through different channels, particularly: (a) email invitation to healthcare professionals and their patients; (b) social medias (Facebook, Twitter, Instagram); (c) mailing lists of universities, national medical associations, and associations of stakeholders (i.e., associations of users/caregivers); and (d) other official websites (i.e., healthcare or welfare authorities’ websites). The online survey was launched through EUSurvey, a web platform promoted by the European Commission, and it took from 15 to 45 min to be filled.

### 2.2. Assessment Tools

Participants to the survey completed an ad-hoc schedule for collecting socio-demographic and clinical information, including the presence of a pre-existing mental or physical disorder, having been infected by COVID-19 or subjected to quarantine, and patterns of internet and web-resources use. The main outcome of this paper, namely the use of mental health resources by the general population, was investigated by two ad-hoc questions (“Since the beginning of the pandemic, did you attend a psychiatric consultation?”, and “Since the beginning of the pandemic, did you attend counselling/psychological consultation?”). Possible answers were “Yes” or “No” for both questions. The two items were collapsed into a single item, considered a proxy for access to mental health resources during the pandemic.

The psychopathological assessment included the Depression, Anxiety, Stress Scale (DASS-21) [50], the Insomnia Severity Index (ISI) [51], the Brief-COPE, the Multidimensional Scale of Perceived Social Support (MSPSS), the UCLA loneliness scale—short version, the Impact of Event Scale (IES)—short version, the Severity of Acute Stress Symptom scale (SASS), the Post-Traumatic Growth Inventory (PTGI)—short form, and the Obsessive-Compulsive Inventory—Revised version (OCI-R). The DASS-21 includes 21 items grouped in three subscales, namely, depression, anxiety, and stress. Each item is rated on a 4-level Likert scale from 0 (never) to 3 (almost always). The DASS-21 total score can be obtained from the sum of the response values of each item and higher scores suggest more severe levels of depressive, anxiety, and stress symptoms [41]. The ISI is a brief instrument assessing different components of insomnia. It includes seven items rated on a 5-level Likert scale, with a total score ranging from 0 to 28 and higher scores indicating higher severity of insomnia [42]. The Brief-COPE consists of 28 items rated on a 4-level Likert scale. Coping strategies are divided in maladaptive (denial, venting, behavioral disengagement, self-blame, self-distraction, and substance abuse) and adaptive (emotional support, use of information, positive reframing, planning, and acceptance) strategies. Two other subscales include religion and humor [52]. The COPE-avoidant score is represented by the mean score at the maladaptive strategies subscales, whilst for obtaining the COPE-approach score the mean of adaptive subscales is considered [53]. The MSPSS consists of 12 items rated on a 7-level Likert scale, which are categorized into three dimensions, namely, family support, support by friends, and support by significant others. Higher scores identify higher levels of perceived support [54]. The UCLA loneliness scale—short version is an instrument conceived to measure feelings of loneliness and social isolation, which is composed of eight items scored on a 4-level Likert scale. Although no clinically meaningful cutoffs have been established for this scale, higher scores account for higher levels of loneliness [55]. The IES—short version assesses psychological reactions in people who have experienced traumatic events, investigating the dimensions of intrusion, avoidance, and arousal disturbances. It consists of 6 items rated on a 5-point Likert scale [56]. The SASS assesses acute traumatic stress-related symptoms and consists of seven items rated on a 5-point scale, with higher scores indicating a greater severity of acute stress disorders [57]. The short form of the Post-Traumatic Growth Inventory (PTGI) is a 10-item assessment instrument subdivided into five dimensions: relating to others, new possibilities, personal strengths, spiritual change, and appreciation of life. Items are scored on a 6-point Likert scale, with higher scores indicating higher levels of post-traumatic growth [58]. The OCI-R is an 18-item assessment instrument exploring the main symptom dimensions of obsessive-compulsive disorder (OCD), including checking, washing, ordering, hoarding, obsessing, and neutralizing. Items are rated on a 5-level Likert scale from 0 to 4 and the total score is calculated by adding all single items. A cutoff of 21 has been established for the diagnosis of OCD [59]. Further measures included in the COMET assessment and described elsewhere [60] were the General Health Questionnaire—12 items version (GHQ) [61], the Connor–Davidson Resilience Scale (CD-RISC), the Suicidal Ideation Attributes Scale (SIDAS) [62], and the Maslach Burnout Inventory (MBI) [63]. The assessment tools included in the COMET study are summarized in Table 1.

### 2.3. Statistical Analysis

Descriptive analyses were carried out in order to evaluate the distribution of sociodemographic and clinical characteristics in the sample. Differences in those accessing mental health services compared to the remaining sample were analyzed using a chi-square test for categorical variables and Student’s *t*-test for continuous variables. Given the large sample size, a parametric technique was chosen due to its sensitivity and since it guarantees sufficient robustness even in case of normality assumption violation [64,65,66]. A multivariate logistic regression model was implemented in order to evaluate the association between use of mental health resources and other significant independent variables, namely, diagnosis of a psychiatric disorder, being a health professional, being unemployed, being in smart-working, scores on the DASS-21, ISI, COPE-avoidant, COPE-approach, MSPSS, and UCLA. The model was adjusted for the period when the interview was administered, for gender, and for age. Odds ratios (OR) with 95% confidence intervals were assessed for observed associations. All tolerance values in the regression analyses were >0.2 and all variance inflation factors were <2, indicating that multicollinearity was not a source of bias in the regression model. Statistical analyses were performed using the Statistical Package for Social Sciences (SPSS) for Windows, version 21. All *p*-values were 2-tailed, and statistical significance was set at *p* < 0.05.

## 3. Results

### 3.1. Sociodemographic and Clinical Characteristics

The whole sample consisted of 20,720 subjects. Eight participants were excluded, since they did not answer the specific questions on the use of mental health services. Therefore, the final sample consisted of 20,712 participants. Among these, 14,712 (*N* = 71%) were females, with a mean age of 40.42 ± 14.32 years. All socio-demographic characteristics of the COMET trial population are described in detail elsewhere [6].

Since the beginning of the pandemic, 1601 (7.7%) participants have had a contact with mental health services. In particular, 1503 subjects (93.9%) requested a psychological assessment and 252 (15.7%) underwent a psychiatric consultation. Moreover, 11.8% (*N* = 189) of participants who accessed mental health services were health professionals; 7.4% (*N* = 119) suffered from a pre-existing mental disorder; 5.9% (*N* = 94) of subjects belonging to this subpopulation lost their job due to the pandemic, while 35.6% (*N* = 570) shifted to smart-working. In the overall sample of subjects referring to mental health services, about one-third of participants were satisfied with their economic condition (*N* = 434, 27.1%) or living condition (*N* = 582, 36.4%). None of the respondents receiving a mental health consultation had spent more time on the Internet since the beginning of the pandemic. Only 1.2% (*N* = 20) of people seen in psychiatric services had been affected by COVID-19, while 4.7% (*N* = 75) of them had been subjected to quarantine measures.

### 3.2. Comparisons between Subjects Referring or Not Referring to Mental Health Services

Unemployment (11.4% vs. 9.1%, *p* = 0.003) and smart-working rates (51.9% vs. 48.6%, *p* = 0.038) were higher in subjects referring to mental health services compared to the remaining sample. No other statistically significant socio-demographic difference was found between the two groups. The use of mental health services did not differ according to the weeks of the lockdown nor to the geographic area of participants.

People accessing mental health services reported higher levels of stress (8.4 ± 3.4 vs. 8.1 ± 3.6, *p* = 0.005), anxiety (4.0 ± 3.5 vs. 3.7 ± 3.4, *p* = 0.001), and depression (6.4 ± 3.7 vs. 6.1 ± 3.8, *p* = 0.001) on the DASS-21 compared to those not accessing those services. Subjects in this subgroup also reported higher levels of insomnia (ISI mean score 7.1 ± 5.4 vs. 6.8 ± 5.2, *p* = 0.009). As for coping strategies, the mean scores of both the COPE-avoidant and COPE-approach subscales were higher in subjects who sought mental healthcare during the lockdown (COPE-avoidant mean score: 4.25 ± 0.8 vs. 3.85 ± 0.8, *p* < 0.001; COPE-approach mean score: 5.68 ± 1 vs. 5.50 ± 1, *p* < 0.001) compared to the remaining sample. Moreover, those people also reported lower levels of perceived social support (MSPSS mean score: 4.9 ± 1.4 vs. 5.3 ± 1.3, *p* < 0.001) and higher levels of perceived loneliness (UCLA mean score: 12.5 ± 3.3 vs. 11.2 ± 3.2, *p* < 0.001) (Table 2).

### 3.3. Factors Associated with the Use of Mental Health Services during the Pandemic

When performing a multivariate logistic regression with the use of mental health resources as a dependent variable, the model (χ^2^(13) = 442.629, *p* < 0.001) explained between 3% and 7.2% of the variance. Variables that showed a significant positive association with the use of mental health services during the pandemic were the UCLA total score (OR 1.079, 95% CI 1.056–1.101, *p* < 0.001), the COPE avoidant (OR 1.586, 95% CI 1.458–1.725, *p* < 0.001) and approach (OR 1.215, 95% CI 1.138–1.299, *p* < 0.001) subscales, and doing smart-working (OR 1.122, 95% CI 0.980–1.285, *p* = 0.095). Being a health care professional (OR 0.841, 95% CI 0.744–1.008, *p* = 0.061) and having higher levels of social support (OR 0.833, 95% CI 0.795–0.873, *p* < 0.001) were protective factors against the likelihood of contacting mental health services (Table 3). These results did not change after controlling for age, gender, and for the period of time when the interview was administered.

## 4. Discussion

To the best of our knowledge, our study is one of the first large-scale studies carried out during the first wave of the COVID-19 pandemic focusing on the use of mental health resources. The main outcome of the study was to evaluate the use of mental health services during the first wave of the COVID-19 pandemic. In our sample, 91.2% of participants were at-risk for developing a mental health problem [60], but only 7.7% consulted a psychologist or a psychiatrist. This finding is in line with a cross-sectional study conducted in France, where only 3.6% of subjects in need for psychological help consulted mental health professionals during the lockdown [67]. These data may to some extent mirror results from the World Health Organization’s (WHO) survey, where 60% of participating countries reported disruptions in mental health services for vulnerable populations, whilst 67% conveyed psychotherapy and counselling services disruption [68]. Furthermore, previous studies described a reduction of psychiatric emergency visits during the first wave of the pandemic [36]. A significant reduction in referrals and self-presentation to community mental health services was also reported in the United Kingdom, without clear evidence of a redistribution of cases among other services [69]. This could be due to both the fear of contagion [70] and the shift towards telemedicine [71,72].

During the first weeks of the pandemic, the reduced access rate to health services was not specific to mental health services, rather it was a global trend [73]. The reorganization of health services and the implementation of extraordinary measures for containing the pandemic may have also had an impact on the access rate, since changes in health resources allocation due to COVID-19 possibly reduced the delivery of specific services, e.g., outpatient visits, to subjects suffering from other diseases [37]. It is noteworthy that Italian data revealed that inpatient admissions were reduced by 17%. In this scenario, digital health interventions were implemented for different medical specialties, but these are still burdened by several matters, such as ethical, legal, and accessibility issues that may account for the underutilization of these resources [74,75]. Telemedicine thus represents a still growing field, which should hopefully be empowered in the near future in order to overcome the above-mentioned shortcomings and reach a higher number of subjects suffering from different conditions.

The effects of the pandemic on vulnerable groups, such as people with pre-existing mental illnesses, are expected to be more severe due to the limited social networks and the presence of several physical comorbidities [76,77,78]. In our sample, only 10.5% of people with pre-existing mental disorders accessed mental health services, which is a much lower rate compared to 25% observed in Canada, both for face-to-face and online consultations [79]. Therefore, our results confirm a reduction in the use of mental health services [80,81]. Moreover, in our sample the presence of a pre-existing mental disorder was not associated with help-seeking behavior, which might be due to the presence of stigmatizing and discriminating attitudes [8,82,83], further nurtured by the fear of the contagion by the virus [84]. There is the need to foster anti-stigma interventions both at the general population level, promoting the public image of psychiatry, as well as at the patient individual level, for challenging internalized stigma [85,86]. Finally, the low access rate to healthcare services found in people with pre-existing mental disorders highlights the need to create specific “soft entry points” for those people, which also represents a high-risk population for the morbidity and mortality related to COVID-19 [69,83,87,88].

Another interesting finding is that unemployment—considered as one of the “proxy” measures of the immediate social consequence of the pandemic—may influence access to mental health services, which is consistent with the finding that economic factors are the main determinants of perceived stress requiring mental health support [67,89]. The pandemic caused an unprecedented socio-economic crisis worldwide, with a shortage of financial resources and increase in unemployment rates. Such psychosocial consequences, as witnessed by previous epidemics, may worsen mental health outcomes, increasing the risk of suicide, and contributing to the adoption of specific risky behaviors, such as alcohol and substance abuse [73,90,91,92,93]. Global health policies should take into account financial aspects as major determinants of mental health and well-being, thus considering economic support as a concrete strategy that could help mitigating the psychiatric consequences of the pandemic.

Among the work-related factors influencing access to mental health services, we found that those practicing smart-working more frequently asked for a visit with a psychiatrist or a psychologist. Smart-working can have negative effects on mental health, as a consequence of reduced physical activity, increased social isolation and perceived loneliness [94]. Furthermore, loneliness and low levels of perceived social support were also significantly associated with access to mental health resources. Loneliness increases the risk of suicidal behaviors and, together with social isolation, is associated with the development of depressive and anxiety symptoms [95,96,97]. Subsequently, loneliness should be monitored in routine clinical practice, not only by healthcare professionals, representing a new strategy for suicide prevention [98,99,100].

Insomnia and sleep disturbances represented a significant predictor of access to mental health care services. This was an expected result considering that sleep disturbances and complaints were highly prevalent during the pandemic and were significantly associated with psychological problems in different populations [101,102,103,104]. Lockdown measures may have altered circadian rhythms, and particularly sleep–wake pattern, through changes in working habits and levels of diurnal physical activity [105]. In fact, sleep disorders were one of the most common causes of psychiatric consultations during the pandemic [106,107]. Therefore, at the population level, preventive educational interventions focusing on the sleep–wake pattern should be provided on a regular basis [108]. Higher levels of depression, anxiety, and stress symptoms were also reported among mental health services users. Of note, depression and anxiety showed a sharp increase among the general population during the first weeks after the pandemic outbreak [3]. Furthermore, depression was hypothesized to be one of the post-COVID-19 features predicting a higher need for psychological support [109].

In our sample, the presence of post-traumatic symptoms was not associated with the probability to access mental health services, although these symptoms are frequent in COVID-19 survivors [110]. It could be that avoidance symptoms experienced by these patients may limit access to healthcare centers [111]. Similarly, obsessive-compulsive symptoms were not associated with access to mental health care, although a worsening of these symptoms was reported after the pandemic onset [112]. Possibly, obsessions concerning hygiene and contamination, that were demonstrated to be particularly prevalent during the pandemic [113], may have limited access to care. Despite this, mental health care among subjects suffering from obsessive-compulsive disorder should be promoted, also considering that treatment adjustment were pointed out as a priority in this population after the CoV-SARS-2 outbreak [114]. Future studies could shed light on the psychopathological dimensions influencing the emergence of psychological distress during the pandemic, but also on attitudes of subjects towards healthcare, such as emotional dysregulation [115], affective temperaments [116], and health anxiety [117].

Not surprisingly, people using adaptive coping strategies were more likely to access mental health services during the lockdown [118,119]. However, it could be that people receiving adequate professional support are more likely to develop adaptive coping strategies [47]. On the other side, the adoption of avoidant coping strategies may influence the development of psychiatric symptoms, indirectly affecting access to mental health care services [120]. Future studies should clarify the relationship between coping strategies and access to mental health service in the pandemic era [83,121].

The main limitations of the study include the use of an online survey and of the snowball sampling procedure, which may have biased the recruitment process and the generalizability of our findings. Moreover, data concerning the attendance to psychiatric and psychological consultations were retrospectively collected and were not cross-checked with official data from specific settings, e.g., emergency department admissions. This also contributed to the lack of information concerning the reason why consultations were requested, preventing researchers from drawing conclusions on causal associations between specific factors, e.g., SARS-CoV-2 infection and economic factors, and access to mental health care.

## 5. Conclusions

The present study highlights that only a minority of subjects suffering from pre-existing mental disorders accessed mental health services during the first wave of the pandemic. Our results suggest that specific strategies should be implemented in order to promote pathways to care in the general population. To this effect, the identification of risk and protective factors could help target interventions to vulnerable groups and enhance a personalized approach to treatments both during and in the aftermath of the pandemic.

## Figures and Tables

**Table 1:** Clinical scales used for psychopathological assessment in the COMET trial.

Assessment Tool	Structure	Considered Time Frame	Scoring
Depression, Anxiety, Stress Scale (DASS-21)	21 items 4-point scale (0–3) Three subscales (depression, anxiety, stress)	Last week	Sum of item scores Higher scores = higher severity
Insomnia Severity Index (ISI)	7 items 5-point scale (0–4)	Last two weeks	Sum of items scores Score ≥ 15 = clinically relevant insomnia
Brief-COPE	21 items 4-point scale Two subscales (approach, avoidant) plus religion and humor	Lifetime	Mean score of the items belonging to each subscale Higher scores = higher tendency to use the coping strategy
Multidimensional Scale of Perceived Social Support (MSPSS)	21 items 7-point scale (1–7) Three dimensions (support from family, friends, significant others)	Lifetime	Mean score of items (both for total score and dimensional scores) Higher scores = higher perceived social support
UCLA loneliness scale	8 items 4-point scale (1–4)	Lifetime	Sum of item scores (range 8–32) Higher scores = higher levels of perceived loneliness
Impact of Event Scale (IES)—short version	6 items 5-point scale (0–4) Three dimensions (intrusion, avoidance, arousal disturbances)	Last week	Mean of item scores Higher scores = higher symptom severity
Severity of Acute Stress Symptom scale (SASS)	9 items 5-point scale (0–4)	Last week	Sum of item scores (also possible to calculate an average score) Higher scores = higher symptom severity
Post-Traumatic Growth Inventory (PTGI)—short form	10 items 6-point scale (0–5) Five dimensions: relating to others, new possibilities, personal strengths, spiritual change, appreciation of life	Lifetime	Sum of item scores Higher scores = higher levels of post-traumatic growth
Obsessive-Compulsive Inventory—Revised version (OCI-R)	18 items 5-point scale (0–4)	Last month	Sum of item scores Score ≥ 21: diagnosis of CD
General Health Questionnaire—12 items (GHQ)	12 items (6 positively worded, 6 negatively worded) 4-point scale (0–3)	Last two weeks	First two types of answers: 0 (positive); last two types of answers: score 1 (negative) Score ≥ 4: probability > 80% of having a mental health problem
Connor-Davidson Resilience Scale (CD-RISC)	10 items 6-point scale (0–5) Five factors: personal competence, high standards, and tenacity; trust in one’s instincts, tolerance of negative affect, and strengthening effects of stress; positive acceptance of change and secure relationships; control; spiritual influences	Last month	Sum of item scores Higher scores = higher levels of resilience
Suicidal Ideation Attributes Scale (SIDAS)	5 items 10-point scale (0–10) Frequency, controllability, closeness to attempt, distress, interference with daily activities	Last month	Sum of item scores (if item 1 is scored 0, all the others are skipped; controllability: reverse score) Higher scores = greater suicidal ideation severity
Maslach Burnout Inventory (MBI)	22 items 7-point scale (0–6) Three subscales (emotional exhaustion, depersonalization, personal accomplishment)	Lifetime (as long as it applies)	Sum of item scores for each subscale (no total score) Higher scores = higher levels of burnout for emotional exhaustion and depersonalization subscales, reverse for personal accomplishment

**Table 2:** Comparison of socio-demographic, clinical and psychopathological characteristics of subjects who attended a psychiatric or psychological visit (mental health users, MHU, *N* = 1601, 7.7%) during the first COVID-19 pandemic wave and those who did not.

	Total Sample (*N*, %)	MHU (*N*, %)	nMHU (*N*, %)	χ^2^	*p*
**Socio-demographic features**					
Female gender	14,712 (71)	1167 (72.9)	13,545 (70.9)	2.822	0.093
High impact region (Emilia-Romagna, Lombardia, Piedmont, Veneto)	6470 (31.2)	514 (32.1)	5956 (31.2)	0.564	0.453
Marital status: single	8092 (39.1)	649 (40.5)	7443 (38.9)	1.505	0.220
Scholarity > 13 years	12,839 (62)	979 (61.1)	11,860 (62.1)	0.480	0.488
Working status: student	4846 (23.4)	384 (24)	4462 (23.3)	0.300	0.584
Working status: unemployed	1924 (9.3)	182 (11.4)	1742 (9.1)	8.631	*0.003*
Smart-working	7085 (48.9)	570 (51.9)	6515 (48.6)	4.322	*0.038*
Health professional	2905 (14)	189 (11.8)	2716 (14.2)	6.897	*0.009*
	**Total sample** (mean, SD)	**MHU** (mean, SD)	**nMHU** (mean, SD)	***t***	***p***
Age	40.42 (14.32)	40.22 (14.40)	40.44 (14.31)	0.584	0.559
Number of cohabitants	2.82 (1.37)	2.86 (1.34)	2.81 (1.37)	−1.281	0.200
	**Total sample** (*N*, %)	**MHU** (*N*, %)	**nMHU** (*N*, %)	**χ^2^**	***p***
**Clinical features**					
Pre-existing psychiatric disorder	1133 (5.5)	119 (7.4)	1014 (5.3)	12.516	*<0.001*
Pre-existing medical illness	3012 (14.5)	247 (15.4)	2765 (14.5)	1.019	0.313
	**Total sample** (mean, SD)	**MHU** (mean, SD)	**nMHU** (mean, SD)	***t***	***p***
Number of hours spent on internet	4.84 (2.30)	4.97 (3.13)	4.82 (2.99)	−1.894	0.058
	**Total sample** (*N*, %)	**MHU** (*N*, %)	**nMHU** (*N*, %)	**χ^2^**	***p***
**Pandemic-related features**					
Survey taken during March/first half of April	15,704 (75.8)	1194 (74.6)	14,510 (75.9)	1.338	0.239
Having themselves/relatives infected or quarantined	1088 (5.3)	87 (5.4)	1001 (5.2)	0.078	0.780
	**Total sample** (*N*, %)	**MHU** (mean, SD)	**nMHU** (mean, SD)	***t***	***p***
**Psychopathological characteristics**					
DASS-21	18.04 (8.40)	18.84 (8.19)	17.98 (8.41)	−4.051	*<0.001*
ISI	6.80 (5.19)	7.15 (5.39)	6.78 (5.17)	−2.650	*0.006*
Brief-COPE avoidant score	3.88 (0.79)	4.25 (0.77)	3.85 (0.79)	−19.828	*<0.001*
Brief-COPE approach score	5.52 (1.07)	5.68 (1.05)	5.50 (1.07)	−6.400	*<0.001*
MSPSS	5.31 (1.36)	4.94 (1.45)	5.34 (1.35)	10.700	*<0.001*
UCLA	11.31 (3.26)	12.55 (3.35)	11.20 (3.23)	−15.961	*<0.001*
SASS	6.03 (4.94)	6.26 (4.99)	6.01 (4.93)	−1.886	0.059
IES-R	1.16 (0.86)	1.14 (0.86)	1.16 (0.86)	0.877	0.381
PTGI	19.02 (11.49)	19.19 (11.27)	19.01 (11.51)	−0.595	0.552
OCI-R	10.77 (8.51)	11.13 (8.82)	10.74 (8.48)	−1.687	0.092

Notes: DASS-21 = Depression, Anxiety, Stress Scale—21 items; IES-R = Impact of Event Scale—short version; ISI = Insomnia Severity Index; MSPSS = Multidimensional Scale of Perceived Social Support; OCI = Obsessive-Compulsive Index—Revised version; PTGI = Post-Traumatic Growth Inventory; SASS = Severity of Acute Stress Symptom scale.

**Table 3:** Logistic regression analyzing factors associated with access to mental health care resources in the study population. The model was corrected for gender, age, and time when the interview was performed.

Variables in Equation	Wald	*p*-Value	OR (95% CI)
COPE avoidant	115.086	<0.001	1.586 (1.458–1.725)
COPE approach	33.305	<0.001	1.215 (1.138–1.299)
Smart-working	2.785	0.095	1.122 (0.980–1.285)
UCLA	51.021	<0.001	1.079 (1.056–1.101)
MSPSS	58.081	<0.001	0.833 (0.795–0.873)
Health professional	3.511	0.061	0.842 (0.704–1.008)

Variables not in equation: DASS-21 total score; ISI total score; working status: unemployed; pre-existing psychiatric disorder. Chi-square: 442.629; *df*: 13; *p* < 0.001.

## Data Availability

The data presented in this study are available on request from the corresponding author.
